# Localized and Excimer Triplet Electronic States of Naphthalene Dimers: A Computational Study

**DOI:** 10.3390/molecules30020298

**Published:** 2025-01-13

**Authors:** Lara Martínez-Fernández, Roberto Improta

**Affiliations:** 1 Departamento de Química Física de Materiales, Instituto de Química Física Blas Cabrera, CSIC (Consejo Superior de Investigaciones Científicas), 28006 Madrid, Spain; lmartinez@iqf.csic.es; 2 Istituto di Biostrutture e Bioimmagini-CNR (IBB-CNR), Via De Amicis 95, I-80145 Napoli, Italy

**Keywords:** DFT calculations, double hybrids, absorption spectra, vibrational effects

## Abstract

We perform DFT calculations with different hybrid (ωB97X-D and M05-2X) and double hybrid (B2PLYP-D3 and ωB2PLYP) functionals to characterize the lowest energy triplet excited states of naphthalene monomer and dimers in different stacking arrangements and to simulate their absorption spectra. We show that both excimer and localized triplet minima exist. In the former, the spin density is delocalized over the two monomers, adopting a face-to-face arrangement with a short inter-molecular distance. In the latter, the spin density is localized on a single naphthalene molecule, and different minima or pseudo-minima are possible, the most stable one corresponding to a slipped parallel arrangement. According to B2PLYP-D3 calculations, excimer minima are the most stable, in line with the indications of ADC(2) studies. However, the relative stability of the minima is reverted when including thermal and vibrational effects. Excimer minima exhibit a very intense absorption spectrum, peaking above 500 nm. The computed absorption spectra of localized minima significantly depend on the stacking geometry and do not coincide with that of isolated naphthalene. Hybrid functionals provide very accurate vibronic absorption spectra for naphthalene monomer, both in the singlet and in the triplet state, but underestimate the stability of the excimer triplet.

## 1. Introduction

When a system containing many interacting chromophores (hereafter, generically, multichromophore assembly, MCA) absorbs light, the resulting excited states can exhibit different degrees of localization, i.e., on single or multiple chromophores. This equilibrium between localization and delocalization rules many processes of biological or technological interest, from photosynthesis [1] to solar energy conversion [2,3,4,5], and it is affected by a subtle balance of many chemical and physical effects (electronic, structural, environmental, etc), whose understanding is fundamental for those applications. In this study, we shall focus on the interplay between delocalized and localized triplet electronic states of stacked naphthalene dimers, an issue that has already attracted significant attention, as witnessed by the large number of studies, both experimental and computational, devoted to elucidating the excited state properties of systems where two naphthalene moieties are covalently linked with different degrees of stacking (see, for example, refs. [6,7,8,9,10,11,12,13,14] and references therein). Thirty years ago, it was shown in several compounds that when two units (dimers) of naphthalene (or some derivatives) were covalently bonded by chemical bridges (referred to as dinaphthyl or naphtalenophanes), their triplet electronic states exhibited absorption or phosphorescence spectra that were quite different with respect to that of the triplet in the monomeric specie [8,13,14,15,16,17]. For example, the strong absorption band appearing at ~420 nm in the T_1_ → T_n_ absorption spectrum of the naphthalene monomer is slightly red-shifted in the dimers, and, especially in the latter, a new absorption band appears above 500 nm [8]. Moreover, the phosphorescence spectra of some of the dinaphthyl are less resolved and red-shifted with respect to that of the low-temperature naphthalene. These spectral properties were assigned to triplet excimer [8,13,14,15,16,17], i.e., an excited state dimer whose stability is larger than the van der Waals ground state. According to these studies, the excimer triplet is delocalized over the two moieties. Thus, we shall use this ‘traditional’ nomenclature, labelling as ‘excimer’ triplet states where the spin density is substantially delocalized in the two rings. In analogy with this interpretation, the weak and shallow absorption band appearing above 500 nm in the triplet absorption spectrum in a concentrated toluene solution of naphthalene has been assigned to the contribution of the excimer [8]. The preferential structure of triplet excimers (e.g., face-to-face, in the following f2f, bent, T-shape, L shape, etc.) [11,12] has also been a matter of debate, and the existence of triplet excimer questioned, the spectral features attributed to excimer being in this cases assigned instead to impurities [18]. The interest in the excited state properties of naphthalene dimers, besides that of the particular compound examined, is motivated by their importance as a model system. Their size and symmetry are ideal for computational studies, making them useful test compounds, besides for methodological developments, to disentangle the different chemical and physical effects ruling singlet and triplet interactions in molecules with extended π systems, as discussed, for example, in refs. [9,11,12,19,20,21,22,23,24] (an exhaustive review of all the contributions falling outside the aim of this study).

As discussed also in the present study, examining different arrangements of the dimer provides the opportunity to get direct insights on how the triplet properties change depending on the electronic coupling between the monomers, which is very large in face-to-face parallel dimer and vanishingly small in ‘perpendicular’ ones. Hence, it has been stated that ‘*the naphthalene dimer in the first triplet excited state is a perfect model system for the investigation and understanding of triplet-triplet interactions between molecules with π-systems*’ [11]. Moreover, naphthalene dimer is the minimal model for stacked polyacenes [25,26,27], a very important family of compounds that are promising components for optoelectronic devices [28,29,30,31], increasing the interest in their studies for nanotechnological applications.

In this study, we shall re-examine the energetic and optical properties of naphthalene and its dimer in the gas phase, examining different stacking arrangements and focusing on their triplet electronic states. In detail, we determine their structural features and assign their absorption spectra by comparing the results obtained with hybrid and double hybrid functionals. We shall characterize the energetics and absorption spectra of a localized triplet for different stacking arrangements. In this way, we shall (i) verify if the mere presence of another closely stacked monomer can modify the spectral features of an isolated naphthalene molecule, (ii) get insights into the interplay between localized and excimer triplet states, and (iii) from the methodological point of view, test the reliability of hybrid density functionals in describing this class of compounds. We highlight that the simulation of the absorption spectra of the different triplet minima is an important contribution to the field. Considering that the emission from triplet electronic states is vanishingly small and that, furthermore, phosphorescence from triplet excimer could be forbidden by symmetry [14], the interpretation of the experimental transient absorption spectra is crucial for the identification of the most stable triplet states and the disentanglement of their dynamic behaviour.

## 2. Results

### 2.1. Naphthalene Monomer

Before tackling the description of the dimers, we shall assess the reliability of our computational approach to predict the electronic spectra of naphthalene monomers in their singlet and triplet electronic states.

#### 2.1.1. Singlet Excited States

In Table 1 we report the lowest singlet vertical excitation energies computed for naphthalene by different electronic methods. Although this paper is focused on the triplet electronic states, it is indeed useful to get some additional insights into the reliability of our computational approach. M05-2X/6-31+G(d,p) and ωB97X-D/6-31+G(d,p) provide the same state ordering of the lowest energy excited states predicted by ADC2 and CC2 calculations, though the stability of the lowest energy ‘dark’ S_1_ 1B_3u_ state (L_b_ according to the Platt’s nomenclature) is underestimated, in line with the trend already evidenced for many density functionals [32]. The energy difference between the two lowest energy excited states computed by B2LYP-D3 (0.27 eV) is, instead, close to that obtained at the ADC2 and CC2 levels, though slightly smaller than that predicted by CASPT2 (0.46 eV) [33], confirming the general reliability of double hybrids functionals for the treatment of acene excited states [34]. In any case, an accurate determination of the lowest excitation energy of naphthalene is not an easy task, as witnessed by the strong dependence of the CASPT2 energies on the size of the active space (a full π-active space is necessary) and the number of orbitals considered to compute dynamical correlation (only 1s orbitals can be frozen) [33]. From the quantitative point of view, M05-2X and TD/ωB97X-D/6-31+G(d,p) calculations provide a very accurate estimate of the adiabatic energy (the quantity available from experiments) of the bright 1B_2u_ state (L_a_ according to the Platt nomenclature), within ~0.1 eV with respect to the experimental estimates (Table 1).

**Scheme 1:**
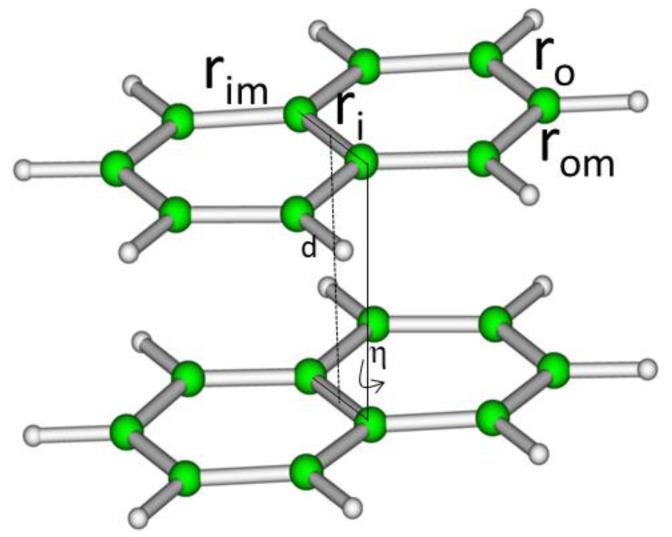
Labelling of the geometry parameters of naphthalene (their values are reported in Tables S1 and S2). η the dihedral formed by **r_i_** bonds, **d** the distance between their midpoints.

**Figure 1:**
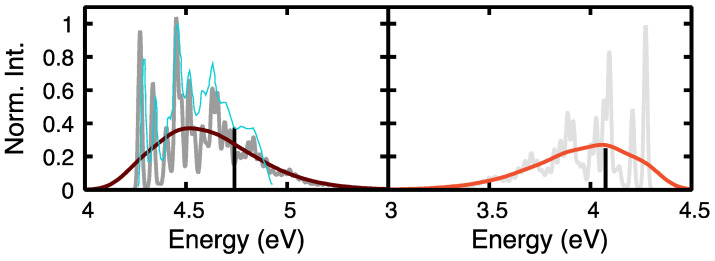
Absorption (**left**) and emission (**right**) spectra of the 1B_2u_ state computed at the ωB97X-D/6-31+G(d,p) level at 300 K using 0.01 eV (grey) or 0.1 eV (reddish) width for the Gaussian used to broaden the stick transition. The vertical absorption and emission energies of the 1B_2u_ state are also reported in black. The experimental absorption spectrum [39] (shifted by −0.15 eV) is shown for comparison in turquoise.

The computed vibrationally resolved absorption spectrum of the L_a_ excited state is also in very good agreement with the available experimental and computational results [35]. As shown in Figure 1(left), the lowest energy vibrational peak is predicted to lie at 4.32 eV, i.e., only ~0.1 eV red-shifted with respect to the experimental one (turquoise line in Figure 1(left)). Our calculations thus attain an accuracy similar to that of CC2 calculations [35]. The vibrational structure of the ground and the excited state are also correctly reproduced (see the spacing and the relative intensity of the different peaks). In addition to the 0-0 transition, in the vibrationally resolved spectrum, we can recognize three main vibrational progressions that are responsible for the most intense peaks. The first, at ~4.33 eV (0–1) ~4.4 eV (0–2), is associated with symmetric ring stretching. The most intense peak, at 4.45 eV, is due to the stretching of the inner central bond (at 1440 cm^−1^) of the naphthalene (r_i_ in Scheme 1), which is elongated in the L_a_ minimum with respect to S_0_. The vibrational progression associated with this mode is responsible for the peaks at 4.62 eV (0–2) and 4.80 eV (0–3). On the blue side of the most intense peak at 4.45 eV, another intense peak is visible (at ~4.48 eV). This is due to the 0–1 transition involving a mode at 1645 cm^−1^, which is associated with the stretching of the ‘external’ C=C bonds (r_o_ in Scheme 1), whose distance is also affected by the electronic transition. The transition to the combination modes of these three vibrational modes is responsible for the main peaks in the spectrum of Figure 1. The geometry shifts associated with these vibrational modes can be visualized in Scheme S1. The red curve of the left panel in Figure 1 reports the computed spectrum simulated by increasing the width of the Gaussian (0.1 eV) function used to convolute any stick transition (to model, for example, the broadening effect due to solvent molecules). Its peak (at ~4.5 eV) is more than 0.2 eV red-shifted with respect to the vertical absorption energy (black vertical line) of the spectroscopic state. This result confirms that the inclusion of vibrational effects leads to a systematic red-shift of the absorption maximum with respect to that obtained by simply applying a phenomenological broadening to the stick transition [38], using a procedure frequently adopted when simulating the absorption spectra, including the contribution of many excited states in multi-chromophore systems, for which a rigorous treatment of vibronic effects is not straightforward.

The spectrum computed at the M052-X level is very similar to the ωB97X-D one (Figure S1), and its accuracy does not significantly change when using the larger def2tzvp basis set (Figure S2).

For what concerns the calculation of the emission spectrum, the inclusion of vibronic effects usually leads to a blue shift of the spectrum with respect to the computed vertical emission energy, but, as shown in Figure 1 (right panel), this effect is less significant [38].

#### 2.1.2. Triplet Excited States

**Figure 2:**
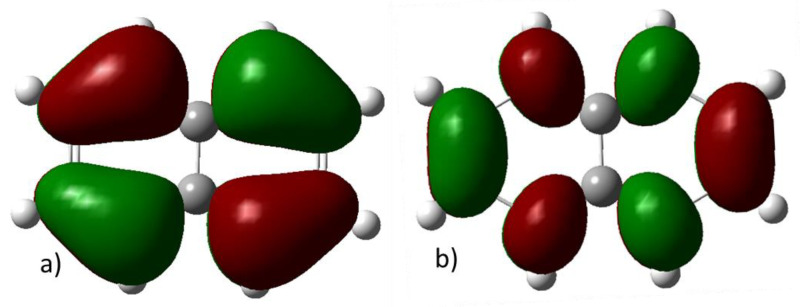
Lowest energy singly occupied molecular orbitals for the T_1_-min of naphthalene. (**a**) Singly occupied molecular orbital (SOMO)-1. (**b**) SOMO.

As a next step, we have characterized the lowest energy triplet electronic state of naphthalene. The lowest energy-occupied orbitals of the triplet minimum are shown in Figure 2, whereas the most important geometry shifts with respect to the S_0_ minimum are reported in Tables S1 and S2 in the Supplementary Materials (the definition of the geometry parameters is in Scheme 1). In Table 2, we report the energy difference between the lowest energy singlet and triplet minima, which, for all the computational methods tested, appear in good agreement with the experimental indications.

**Figure 3:**
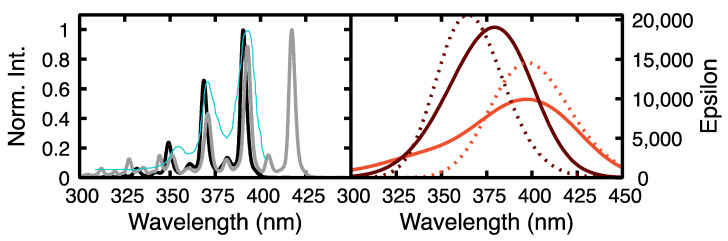
(**Left**) Vibrationally resolved absorption spectra computed for the T_1_ triplet minima of naphthalene in the gas phase at the ωB97X-D/6-31+G(d,p) level (broadening of 0.01 eV applied to each vibronic transition). The intensity of the TDA spectrum (black) is normalized to that of the TD (grey) one. The experimental transient absorption spectra of naphthalene in 2-propanol after photosensitization with benzophenone are shown for comparison in turquoise [42] (shifted by +0.15 eV). (**Right**) Vibronic spectra (simulated by applying a broadening of 0.15 eV to each vibronic transition; solid lines) are compared with the spectrum obtained by convoluting the electronic stick transition with a Gaussian with hwhm = 0.2 eV (dashed lines) at both TDA (dark red) and TD (red) levels.

In Table 3, we report the VAE computed for the lowest energy triplet by different electronic methods, both at the TD and the TDA levels. T_2_ and T_5_ can be described as the symmetric and antisymmetric combination of the SOMO(α) → LUMO+1(α) and SOMO(β) → LUMO+1(β) excitations. T_3_ and T_4_ derive instead from the symmetric and antisymmetric combination of the SOMO(α) → LUMO(α) and SOMO(β) → LUMO (β) excitations. According to both TD and TDA calculations, the lowest energy (and only) triplet bright state correspond to T_5_ (or to T_6_ at the TDA/B2PLYP-D3 level), which we have used to compute the triplet absorption spectrum of naphthalene (Figure 3) using different approaches. On the left, the vibrationally resolved spectra, computed both at the ωB97X-D/6-31+G(d,p) TD and TDA levels, are shown, considering the T_1_ → T_5_ transition. The experimental transient absorption spectrum of naphthalene in its triplet state in apolar solution is also shown.

Three peaks of decreasing intensity are clearly recognizable in the experimental spectrum at ~415 nm, ~390 nm, and ~370 nm. The positions of these three peaks are reproduced almost quantitatively by TD calculations, but the relative intensity of the low-energy, most prominent peak is slightly underestimated. The relative intensities of the three peaks are instead perfectly reproduced at the TDA level, but their position is uniformly slightly blue-shifted with respect to the experiments. However, as shown in Figure S3, a part of this error could be due to the absence of solvent effect, as the spectrum computed in 2-propanol is red-shifted by ~15 nm. Also, in this case, the inclusion of vibronic effects is essential to reproduce not only the line shape but also the position of the spectrum. At the TDA level, the vertical absorption energy T_1_ → T_5_ (responsible for the lowest energy absorption band) falls at 3.40 eV (365 nm), i.e., ~0.2 eV (~25 nm) blue-shifted with respect to the most intense peak of the vibrationally resolved computed spectrum.

The two strong bands appearing in the experimental spectrum below the 0-0 transition, i.e., at 370 and 390 nm, are due to two vibrational progressions on two ring stretching modes. One, falling at 1465 cm^−1^ at the TDA level, can be described as a symmetric stretching mode of r_im_ CC bonds (Scheme 1). The other one, at 1558 cm^−1^, as a symmetric stretching of the r_i_ and r_o_ CC bonds.

Most of the experimentally available transient absorption spectra, especially those in concentrated solution, are less resolved than those reported in Figure 3. Moreover, the application of the most accurate procedures for the calculation of vibronic spectra is not straightforward in the presence of multiple electronic bright transitions, as we find for stacked dimers (as discussed below). As a consequence, we assess the performance of a simpler procedure, often adopted to simulate the absorption spectra of large systems, by broadening the stick transitions with a phenomenological Gaussian. In the right panel of Figure 3, the vibronic transitions reported are broadened with a Gaussian with half width half maximum of 0.15 eV, which is sufficient to wash any resolution. They are reproduced sufficiently well by the spectra based on the stick transitions by using a broadening of 0.2 or 0.25 eV (Figure S3), at least for the scope of the present paper. Thus, we resort to this simpler procedure, which underestimates the width of the spectrum and, in the case of TDA, leads to a weak blue shift (10~20 nm) of the maximum.

### 2.2. Naphthalene Dimers

#### 2.2.1. Triplet Minima and Stabilities

**Figure 4:**
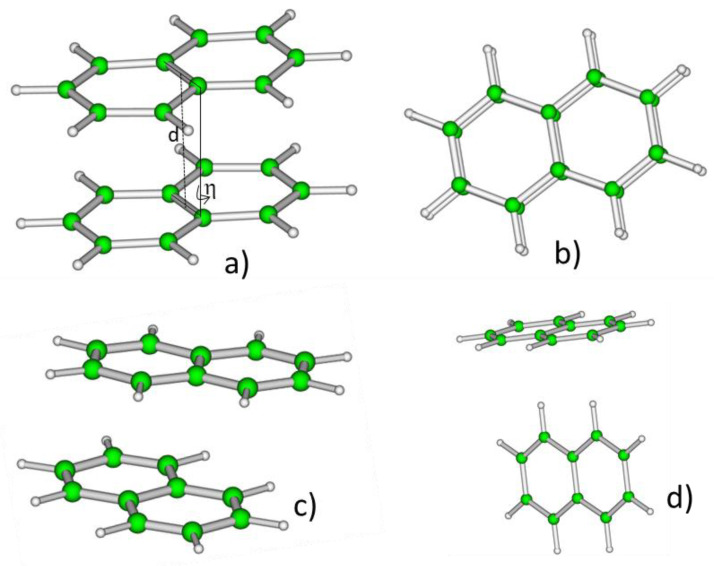
Schematic description of a naphthalene dimer in different arrangements: (**a**) face-to-face parallel (side view), (**b**) face-to-face parallel (top view), (**c**) slipped parallel, and (**d**) T.

**Figure 5:**
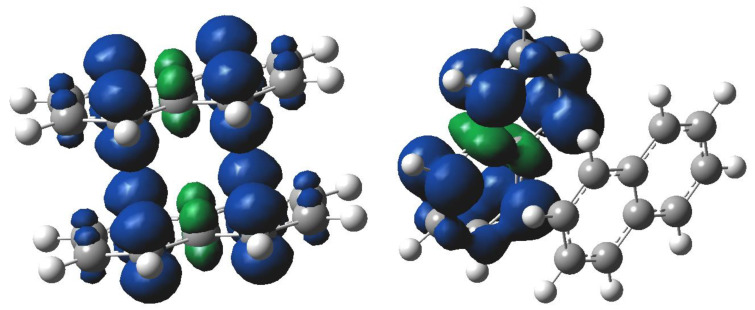
Mulliken spin density computed at the B2LYP-D3/6-31+G(d,p) level for T_1_-exc-min (**left**) and T_1_-loc-min (**right**). Colour code: green, negative spin density; blue, positive spin density.

**Figure 6:**
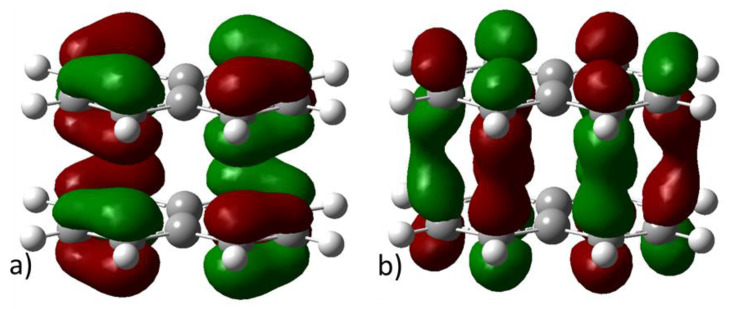
Lowest energy single occupied molecular orbitals for T_1_-exc-min. (**a**) SOMO-1. (**b**) SOMO.

We optimized the lowest energy triplet states for a naphthalene dimer in different stacking arrangements (Figure 4) using the same approaches explored in the study of the monomer. In the following section, we used ωB97X-D and B2LYP-D3 as reference methods. We succeeded in locating the minima of two different triplet electronic states. In one of them, the triplet is equally delocalized over the two naphthalene rings (Figure 5 left panel), the triplet ‘excimer’ introduced above, and we label it T_1_-exc (and its minimum, T_1_-exc-min). As shown in Figure 6, the two lowest energy singly occupied MOs are perfectly delocalized over two monomers, as found in the pioneering study of Pabst et al., based on ADC(2) calculations [11]. In T_1_-exc-min, the monomers adopt a perfect ‘face-to-face’ parallel arrangement, and two moieties are much closer than in the ground state minimum, with d decreasing by 0.4~0.5 Å to a value of 3.17~3.18 Å (depending on the electronic method). The SOMO has a significant bonding character between pairs of atoms belonging to different rings, leading to a much closer approach between the two monomers. The intra-monomer structural parameters (Tables S1 and S2) are instead intermediate between those typical of the singlet and those of a triplet, in line with a picture of spin density delocalized over the two rings. The structural features of T_1_-exc-min predicted by ωB97X-D and B2LYP-D3 are very similar, and they have an extremely limited dependence on the size of the basis set. For example, B2PLYP-D3 predict the same **d** value by using either 6-31+G(d,p) or the cc-pvtz basis set. The **d** value is slightly larger than that predicted by ADC2 calculations, 3.08 Å.

In the other triplet, the analysis of the Molecular Orbitals (MOs) and of the Mulliken spin densities (Figure 5 right panel and Figure S4) indicate instead that the triplet is essentially localized over a single naphthalene moiety. We label this excited state as T_1_-loc (Figure 5), as ^3^N the naphthalene molecule where the triplet is localized, and as ^1^N the one in the singlet state. For T_1_-loc, different stationary points are possible, as happens for a stacked dimer in S_0_ [22]. A complete characterization of the Potential Energy Surface (PES) of the lowest energy adiabatic triplet (locating all the minima using reliable automatic procedures) [43] is outside the scope of the present study. We focus on three representative structures, enabling us to explore different degrees of electronic coupling between the monomers. In the most stable localized minimum (hereafter T_1_-loc-min), the two monomers exhibit a slipped parallel arrangement (Figure 4c); however, perfect ‘face-to-face’ arrangement or T-shaped conformation with two ‘short’ perpendicular molecular axes (Figure 4d) are also possible. On the other hand, these two latter structures (hereafter T_1_-loc-f2f and T_1_-loc-T) exhibit a small negative frequency (<50 cm^−1^) and are not true minima of the T_1_-loc PES, at least by using ωB97X-D or M05-2X functionals. B2LYP-D3 and ωB97X-D calculations provide a similar picture, but T_1_-loc-f2f is a real minimum in the PES according to the former method, and, actually, it is slightly more stable than T_1_-loc-min, at least at the electronic level.

The geometries of the naphthalene molecules in the different T_1_-loc minima are very similar. ^3^N and ^1^N have structures that are very similar to that of isolated naphthalene in its triplet and singlet states, respectively (Tables S1 and S2). The effect of the inter-monomer stacking on the geometries is thus quite small. Actually, though the **d** value in T_1_-loc-min is significantly larger than in T_1_-loc-f2f, the distance between the two rings in the two minima is similar, i.e., 3.5~3.6 Å. Also, in T_1_-loc-min, the two monomers are not perfectly parallel.

As shown in Table 4 and Table S3, the relative stability of T_1_-loc-min and T_1_-exc-min minima depends on the adopted computational approach. Hybrid functionals (also including long-range corrections and empirical corrections) predict that T_1_-loc-min is ~0.3 eV more stable than T_1_-exc-min. Double hybrid functionals favour T_1_-exc-min, which, according to B2LYP-D3, is indeed the most stable triplet minimum, with its relative stability increasing with the size of the basis set. According to ADC(2)/cc-pvtz calculations, when BSSE is considered, T_1_-exc-min is more stable than T_1_-loc-T by 0.29 eV [11]. No mention is made in that study about the possibility of localized minima where two monomers are parallel, which are instead more stable according to hybrid and double hybrid functionals. On balance, we can estimate that when compared with ADC(2), the stability of T_1_-exc-min is underestimated by 0.1~0.2 eV (depending on the basis set adopted) by B2PLYP-D3 calculations.

Interestingly, the energy ordering obtained at the ωB2PLYP/6-31+G(d,p) double hybrid level is similar to that provided by hybrid functionals. T_1_-loc-min is more stable than T_1_-loc-f2f by only 0.02 eV and T_1_-exc-min by 0.43 eV. This result is partially due to the absence in ωB2PLYP of empirical terms describing the dispersion interactions, which are expected to be important for the closely stacked T_1_-exc-min. In order to corroborate this hypothesis, we performed some test calculations at the B2PLYP/6-31+G(d,p) level. This method predicts that T_1_-loc-min is more stable than T_1_-exc-min by 0.1 eV, i.e., this latter minimum is relatively destabilized by 0.15 eV when D3 terms are not included. In this latter case, **d** in T_1_-exc-min increases by 0.1 Å with respect to the minimum optimized at the B2PLYP-D3/6-31+G(d,p) level, decreasing the relative stability of the excimer minimum.

The relative stability of the different minima is significantly affected when vibrational and entropic corrections (at the harmonic level) are included. Independently of the electronic methods adopted, T_1_-loc-min is relatively stabilized (by ≥0.2 eV) over T_1_-exc-min. As a consequence, when their free energy is considered, T_1_-loc-min is the most stable triplet minimum, also at the B2LYP-D3 level. This result can be associated with the different features of the PES associated with the triplet different minima. In T_1_-exc-min, the two bases are closely stacked and quite strongly bonded. In T_1_-loc-min, the PES is much shallower than in T_1_-exc. We find many arrangements with very similar energies (even f2f), and there is a larger number of low-energy large amplitude vibrational motions, providing a possible rationale for the free energy stabilization of T_1_-loc.

In summary, the triplet minima of a stacked naphthalene dimer can be classified into two families: localized and delocalized. From the purely electronic point of view, the latter (whose relative stability is underestimated by hybrid density functional) are more stable. In addition, we here also show that localized triplet minima are stable also for parallel arrangements of the monomers, and they are relatively strongly favoured by entropic and vibrational effects.

#### 2.2.2. Absorption Spectra 

**Figure 7:**
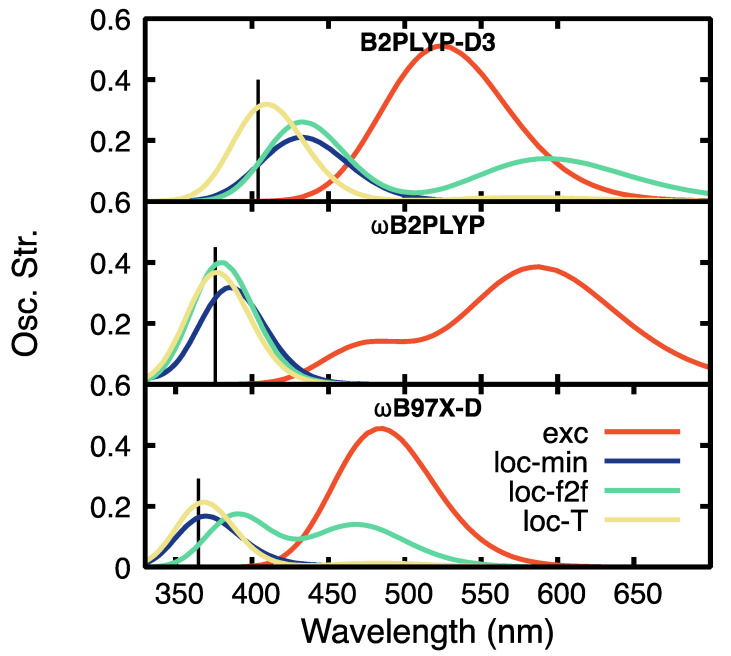
TDA spectra computed at the triplet minima using three different electronic methods. 6-31+G(d,p) basis set. Spectrum obtained by convoluting the electronic stick transitions with a Gaussian with hwhm = 0.2 eV. The vertical bright transitions predicted for the monomer are reported in black.

As the next step of the study, we simulated the absorption spectra of the different triplet minima discussed in the previous section. In Table S4, we report their lowest energy excited states according to different computational approaches, whereas the computed spectra are reported in Figure 7.

In T_1_-exc-min, the lowest energy excited states involve frontier orbitals delocalized over the two monomers. Two excited states are derived from the symmetric and antisymmetric combinations of SOMO(α) → LUMO(α) and SOMO(β) → LUMO(β) excitations. One (T_2_) is completely dark and falls at very low energy (below 0.75 eV), whereas the other, corresponding to T_6_, gives rise to an extremely intense band, with the maximum at ~550 nm or 500 nm (at the ωB97X-D level). We find another bright transition, though less intense, slightly above the main absorption peak. It is associated with T_8_, with SOMO(α) → LUMO+2(α) and SOMO-2(β) → LUMO(β) as the main contributions. Also, this state has a low energy ‘dark’ counterpart (T_4_). The picture provided by B2PLYP-D3 and ωB97X-D calculations fully agrees with that of ADC2 calculations, with two close-lying very intense transitions at 2.5~2.6 eV, giving rise to a strong band at ~500 nm. A similar qualitative picture is also obtained by using ωB2PLYP, with two intense electronic transitions above 450 nm. In this case, the two bright states are more distant, resulting in a broad absorption band with a maximum at ~600 nm and a shoulder at ~500 nm.

**Figure 8:**
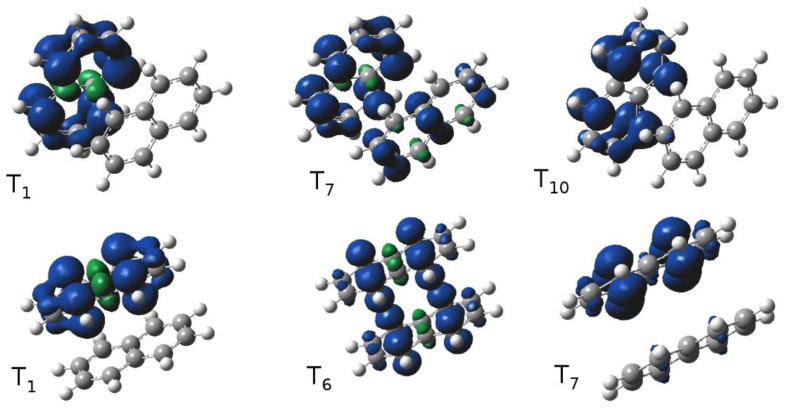
Spin density (threshold ≥ 0.004 a.u.) associated with different triplet excited states in T_1_-loc-min (up) and T_1_-loc-f2f according to TDA/ωB97X-D/6-31+G(d,p) calculations. Positive spin density is depicted in blue, negative in green.

For the three loc minima, the spectra predicted by ωB97X-D significantly depend on the stacking geometry. The spectrum of T_1_-loc-min, though its maximum falls at ~400 nm, is different from that of the monomer. The three lowest excited states (i.e., T_2_, T_3_, and T_4_) correspond to dark transitions that are very similar to that predicted for isolated naphthalene. Then, we find several transitions with significant intermolecular Charge Transfer (CT) character. T_5_ can be described as a ^3^N SOMO(α) → ^1^N LUMO(α) transition, whereas T_6_ has a large ^1^N SOMO(β) → ^3^N LUMO(β) contribution. T_7_, though with a significant ^1^N SOMO-1(β) → ^3^N LUMO(β) contribution, is mixed with the bright transition localized on ^3^N and thus acquires some oscillator strength (Figure 8). T_8_ is localized on ^1^N, and, essentially, corresponds to the vertical transition to the lowest energy triplet states of naphthalene. T_9_ is similar to T_5_, i.e., with quite a large CT character (in this case, with a significant ^3^N SOMO(α) → ^1^N LUMO+1(α) contribution); however, T_7_ has some oscillator strength. Finally, T_10_ is very similar to the lowest energy bright state in the triplet monomer, and the contribution of ^1^N is extremely small (Figure 8). The electronic transitions in T_1_-loc-T are more similar to those of isolated naphthalene, as the electronic coupling between the MOs of ^3^N and ^1^N are smaller than in the stacked system. In T_1_-loc-f2f, we find two bright excited states (corresponding to T_6_ and T_7_) that are similar to the T_7_ and T_10_ states just described for T_1_-loc-min. However, due to the different stacking arrangements, the mixing between the MOs of ^3^N and ^1^N is larger. As a consequence, although in the lowest energy triplet, the spin density is fully localized on ^3^N as in T_1_-loc-f2f (Figure 8), in T_6_ and T_7_, the contribution of ^1^N is significant. As a consequence, the related transitions are red-shifted with respect to what happens in T_1_-loc-min, and the lowest energy one acquires a pretty large oscillator strength, being responsible for the band appearing above 500 nm.

The analysis of the B2PLYP-D3 spectra provides a picture fully consistent with that described above obtained with the ωB97X-D method. The absorption maximum is always lightly red-shifted with respect to the isolated monomer, with the amount of the shift being larger for T_1_-loc and T_1_-loc-f2f. For this latter arrangement, the presence of a fairly intense low-energy transition is also predicted. TDA-ωB2PLYP double hybrid spectra are fairly similar to the B2PLYP-D3 and ωB97X-D ones, but some differences arise. The maximum of the absorption spectrum is indeed still red-shifted with respect to the monomer, but the relevance of the red-wing tail is smaller than that obtained with two other methods. For T_1_-loc-f2f, for example, one weak transition is still predicted to fall on the red of the main electronic transitions, but the energy gap is only 0.01 eV, and its intensity is small (Table S4); therefore, it is buried in the main absorption band.

## 3. Computational Details

Methods: We shall resort to Density Functional Theory (DFT) calculations, adopting both meta-GGA M05-2X [44] and range separated ωB97X-D [45] hybrid functionals (here we refer to these as hybrid density functionals), which provide a fairly reliable description of charge transfer excited states and have already been profitably adopted to study closely stacked systems [46,47], and double hybrid BP2LYP-D3 [48,49] and ωBP2LYP [50] functionals. Double hybrid functionals have recently emerged as very promising tools for the calculation of the excited state properties of several classes of compounds [32,34,51,52,53], such as acenes, which are instead problematic for many density functionals. The spectra have been computed both at the TD level and using the Tamm-Dancoff approximation (TDA), which, in many systems, has been shown to provide a more balanced treatment of singlet and triplet excited states.

Approach: (1) Monomer: we first optimized the ground state of the naphthalene monomer, on top of which we computed the singlet and triplet states, and in a second step, we optimized the S_1_ and T_1_ minima and computed their emission spectra. (2) Dimer: we focus only on the characterization of the triplet manifold, locating different minima whose absorption spectrum was then simulated.

Spectra simulations: (1) Monomer: We resorted to two different approaches. (1a) Vibrationally resolved spectra have been computed by using the FCClasses3 program [54], whose application is not always straightforward when it is necessary to consider the contribution of multiple excited states. To do so, at the corresponding geometry, the frequencies of both ground and excited states are computed, and then the spectra were simulated based on the Adiabatic Hessian approximation [54,55,56]. (1b) We also simulated the spectra with a simple procedure based on the broadening of the stick electronic transitions with a phenomenological Gaussian of a certain half width half maximum (hwhm). We checked, then, that this procedure provides spectral line shapes in good agreement with the experimental one and to the reference computed with FCClasses3. (2) Dimer: the absorption spectra of the dimers have, thus, been simulated by resorting to the simpler procedure by broadening the stick transitions with a phenomenological Gaussian, with hwhm = 0.2 eV.

All the calculations were performed using the Gaussian16 [57] and Orca5.0 [58] programs. If not otherwise specified, geometry optimizations, frequency calculations, and excited state hybrid calculations were performed using the Gaussian16 package, with its default input values, whereas double hybrid excited states were computed using the Orca5.0 package (see Supplementary Materials). Spin density was computed using the Gaussian16 package and visualized using the Gaussview5 program [59].

## 4. Discussion and Conclusions

In this study, we have analysed the lowest energy triplet excited states of naphthalene monomers and dimers in the gas phase, simulating the absorption spectra of different stacking arrangements by integrating DFT and TDA-DFT calculations with hybrid and double hybrids functionals. We have also preliminary checked the accuracy of our computational approach on the lowest energy singlet excited states of the monomer, which is a classic example of the possible failures of many hybrid DFT functionals that often revert their relative stability [32].

*Singlet excited states of naphthalene:* The two functionals examined (ωB97X-D and M05-2X) correctly predict that in the FC region, the dark L_b_ state is more stable than the bright L_a_ state; however, the predicted energy difference is too small, i.e., <0.1 eV. Consequently, the adiabatic energy difference of L_a_ is smaller than that of L_b_, which is in disagreement with the experimental indications. Double hybrid calculations are instead confirmed to provide a L_a_-L_b_ energy gap very close to that of other WF-based methods [33], in good agreement with other experiments [36,37]. It is important to highlight, on the other hand, that both ωB97X-D and M05-2X provide an extremely accurate description of the energy and structure of L_a_; the computed vibronic spectrum is almost superimposable to the experimental one, confirming that the simple analysis of the vertical absorption energies is often not sufficient to assess the accuracy of a computational method [38]. L_a_ and L_b_ are expected to be vibronically coupled (as revealed by the fluorescence excitation spectrum of naphthalene in a supersonic jet) [60], and many intra-molecular vibrations could induce their mixing, suggesting that a purely static description of these two states could have significant limitations, and the usefulness of dynamical studies on this issue [61,62,63,64,65,66,67].

*Triplet excited states of naphthalene:* DFT and double hybrid calculations accurately reproduce the experimental singlet/triplet energy gap and the characteristic band at ~400 nm present in the experimental absorption spectrum of the lowest energy triplet. The relative intensity of the main experimental vibrational peaks in the triplet absorption spectrum is well reproduced by TDA/ωB97X-D/6-31+G(d,p) calculations, indicating that this computationally inexpensive method provides an accurate description of the structural shifts induced by the electronic transition to the lowest energy bright excited triplet states (T_5_ or T_6_ depending on the electronic method). From the quantitative point of view, TDA-B2PLYP-D3 vertical absorption energy is closer to the experimental maximum (420 nm in toluene) [8]. However, considering the contribution of vibronic and solvent effects, and that TD spectra are red-shifted with respect to TDA, ωB97X-D and ωB2PLYP deliver spectra of similar accuracy compared with B2PLYP-D3.

*Triplet excited states of naphthalene dimers:* We characterized the lowest energy triplet electronic states for naphthalene dimers in different stacking geometries. We localized an excimer (T_1_-exc) minimum for the dimer, where the two rings adopt a perfect face-to-face geometry, the intermolecular distance strongly decreases, and the spin density is delocalized over the two monomers. Confirming the indications of a previous ADC(2) study [35], this arrangement thus appears to be the most stable one for an excimer minimum. We also succeeded in localizing minima or pseudo minima (T_1_-loc), where the spin density is essentially localized on a single monomer (^3^N), not only for a T-shaped stacking arrangement but also for parallelly stacked naphthalene molecules. We located two ‘parallel’ localized minima, slipped parallel and f2f, with very similar stabilities. The former is more stable according to hybrid functionals (according to which f2f is not a real minimum), and the latter is more stable according to double hybrids. Actually, it is not surprising that additional local minima are present on the PES of T_1_-loc, in analogy with what happens for two naphthalene molecules in the ground electronic singlet state. The relative stability of these minima depends on the electronic method. B2PLYP-D3 double hybrid functional indicate that it is T_1_-exc-min > T_1_-loc-f2f ~ T_1_-loc-min > T_1_-loc-2T. According to hybrid functionals, it is instead T_1_-loc-min > T_1_-loc-f2f > T_1_-loc-2T > T_1_-exc-min. Our study, however, indicates that electronic interactions are not the only contribution to be considered. Likely due to very different features of T_1_-loc and T_1_-exc minima, the inclusion of vibrational and entropic effects can indeed completely revert the relative stability of the triplet minima. T_1_-loc-min is also predicted to be the lowest free energy minimum at the B2PLYP-D3 level. It is clear that this estimate can be considered only a starting point, considering that a harmonic treatment of low-energy large-amplitude vibrational modes, which are in a larger number for the shallower T_1_-loc-minimum, is surely an approximation. Moreover, B2PLYP-D3 slightly underestimates the relative stability of T_1_-exc-min with respect to ADC(2). Notwithstanding all these caveats, our calculations clearly indicate that in the gas phase, a significant part of the triplet naphthalene dimers are localized on a single monomer. The smaller tendency of triplet electronic states, when compared to singlet state, to give rise to excimers in naphthalene dimers has already been discussed [68]. An interesting recent theoretical study of oligoacene aggregates, including naphthalene, also indicates that CT contributions in triplet exciton states are less important than in singlet excitons [9,11,12,19,20,21,22,23,24], and this effect could play a role in the stabilization of excimeric states and in other molecular aggregates [10,69].

*Beyond the dimer:* It is likely that excimer and localized triplets can be strongly vibronically coupled, at least for some stacking geometry, e.g., f2f. In this arrangement, the variation of the inter-monomer distance would induce some population transfer between T_1_-loc and T_1_-exc. Considering also the large number of possible T_1_-loc minima (T_1_-loc-min and T_1_-loc-f2f are practically isoenergetic), the most likely scenario for situations where a large population of interacting naphthalene molecules is present (as in the highly concentrated solutions) different triplets, essentially localized but where excimer or partially localized triplets, are present, in different stacking arrangement. In this respect, another interesting outcome of this study concerns the simulation of the absorption spectra of the different triplet minima. In T_1_-loc minima, in addition to a ‘monomer-like’ transition at ~400, quite similar to that typical of isolated naphthalene, we find two additional weaker transitions on the red wing of the main absorption band, also involving the stacked naphthalene molecule. The position and the intensity of these transitions depend on the stacking geometry of the dimer. For T_1_-loc-min, they simply produce a weak red tail in the spectrum; on the other hand, for T_1_-loc-f2f, an intense band appears just below 500 nm, extending until 600 nm. As a consequence, the band appearing in the low-energy tail in concentrated naphthalene solution in toluene could not be (exclusively) due to a small population of excimers (which would induce an extremely intense absorption band at 500 nm) but simply to the contribution of T_1_-loc-f2f dimers. Confirming the indications provided by our parallel study of covalently bonded naphthalene dimers in closely stacked systems, localized excited states can have quite different spectral features with respect to the isolated molecules.

Finally, our study provides some indications of the accuracy of the different functionals adopted. Considering the indications of ADC(2) calculations, which predict that T_1_-exc-min is 0.3 eV more stable than T_1_-loc-2T, it is clear that hybrid functionals (which give the opposite results) significantly underestimate the relative stability of the excimer minima, and this limitation should be seriously considered in the study of stacked acenes. On the other hand, ωB2PLYP predicts the same energy ordering as hybrid functionals, which is different to that obtained with B2PLYP–D3. This outcome indicates the importance (confirmed by B2PLYP test calculations) of an accurate description of dispersion interactions. Excimer minima are stabilized by very short inter-monomer distances for which a balanced treatment of dispersion/repulsion terms is important.

## Figures and Tables

**Table 1:** Vertical absorption energies (in eV) and oscillator strength (in parentheses) for the three lowest energy singlet excited electronic states of naphthalene in the gas phase.

	M05-2X 6-31+G(d,p)	ωB97X-D 6-31+G(d,p)	M05-2X def2tzvp	B2PLYP-D3 6-31+G(d,p)	PBE-QIDH ^a^ def2tzvp	ADC2 ^b^	CC2 ^b^	CASPT2 ^c^ cc-pvdz	Exp
S_1_ 1B_3u_	4.79 (0.00) 4.48 ^d^	4.66 (0.00) 4.36 ^d^	4.80 (0.00)	4.43 (0.00)	4.64	4.50 (0.00)	4.49 (0.00)	3.99	4.03 ^e^ 3.98 ^f^
S_2_ 1B_2u_	4.82 (0.08) 4.31 ^d^	4.74 (0.08) 4.26 ^d^	4.80 (0.08)	4.70 (0.15)	4.97	4.87 (0.09)	4.89 (0.08)	4.45	4.38 ^e^ 4.34 ^f^
S_3_ 2B_3u_	6.15 (1.34)	6.11 (1.33)	6.21 (1.34)			6.20 (1.63)	6.22 (1.44)		

(^a^) From ref. [34], (^b^) def2tzvp basis set from ref. [35], (^c^) from ref. [33], (^d^) adiabatic energy difference, (^e^) adiabatic energy difference in the gas phase [36], and (^f^) adiabatic energy difference in neon matrix [37].

**Table 2:** Energy difference (in eV) between the lowest energy singlet and triplet minima for naphthalene monomers, according to different electronic methods.

	Electronic	Sum Electronic and Zero Point Energy	ΔG
M05-2X/6-31+G(d,p)	2.90	2.73	2.69
M05-2X/aug-cc-pvtz	2.90	2.74	2.70
ωB97X-D/6-31+G(d,p)	2.82	2.66	2.62
ωB97X-D/aug-cc-pvtz	2.83	2.67	2.62
B2PLYP-D3/6-31+G(d,p)	2.88	2.75	2.70
B2PLYP-D3/aug-cc-pvtz	2.88	2.76	2.72

Exp: 21,186 (2.63 eV) cm^−1^ (0.0 transition energy) [40,41].

**Table 3:** Vertical absorption energies in eV (and oscillator strength in parenthesis) for naphthalene triplet states in the gas phase.

Naphthalene	ωB97X-D 6-31+G(d,p)	M05-2X 6-31+G(d,p)	ωB97X-D def2tzvp	B2PLYP-D3 6-31+G(d,p)
TD				
T_2_	1.49 (0.00)	1.41 (0.00)	1.53 (0.00)	1.29 (0.00)
T_3_	1.63 (0.00)	1.61 (0.00)	1.64 (0.00)	1.56 (0.00)
T_4_	2.18 (0.00)	2.18 (0.00)	2.18 (0.00)	2.21 (0.00)
T_5_	3.11 (0.21)	3.12 (0.21)	3.15 (0.20)	2.89 (0.31)
T_6_	3.24 (0.00)	3.26 (0.01)	3.30 (0.00)	3.02 (0.00)
TDA				
T_2_	1.77 (0.00)	1.75 (0.00)	1.81 (0.00)	1.71 (0.00)
T_3_	1.80 (0.00)	1.77 (0.00)	1.82 (0.00)	1.75 (0.00)
T_4_	2.26 (0.00)	2.28 (0.00)	2.26 (0.00)	2.26 (0.00)
T_5_ T_6_	3.40 (0.29) 3.45 (0.00)	3.40 (0.30) 3.46 (0.01)	3.45 (0.29) 3.50 (0.00)	3.03 (0.00) 3.07 (0.40)

**Table 4:** Relative energy (in eV), with respect to T_1_-exc-min, for different T_1_-loc stationary points located for the naphthalene dimer in the gas phase.

	T_1_-loc-min		T_1_-loc-f2f	T_1_-loc-T
	Electronic Energy	Free Energy	Electronic Energy	Electronic Energy
ωB97X-D/6-31+G(d,p)	−0.35	−0.56	−0.33	−0.26
ωB97X-D/def2tzvp	−0.35	−0.57	−0.34	−0.27
ωB97X-D/aug-cc-pvtz	−0.35 ^a^	−0.58	−0.35	−0.27
B2PLYP-D3/6-31+G(d,p)	+0.05 ^b^	−0.18	+0.025 *	+0.15
B2PLYP-D3/def2tzvp	+0.095		+0.06	+0.19

* real minimum, free energy −0.12. (^a^) BSSE corrected −0.35; (^b^) BSSE corrected +0.03.

## Data Availability

The data that support the findings of this study are reported in the main text and in SI of this article and are available on request from the corresponding author. The funders had no role in the design of the study; in the collection, analyses, or interpretation of data; in the writing of the manuscript; or in the decision to publish the results.

## Supplementary Materials

The following supporting information can be downloaded at: https://www.mdpi.com/article/10.3390/molecules30020298/s1. Scheme S1: Schematic depiction of two relevant vibrational modes for the triplet spectrum of naphthalene monomer, Figure S1: Absorption and emission spectra of naphthalene computed with M05-2X functional, Figure S2: Absorption spectrum of naphthalene computed with def2tzvp basis set, Figure S3: Triplet absorption spectrum of naphthalene computed in 2-propanol and two different hwhm, Tables S1 and S2: Main geometrical parameters, Figure S4: Mulliken spin population computed at the B2LYP-D3/6-31+G(d,p) level, Table S3: Relative energies of the triplet minima with different methods, Table S4: Lowest energy transitions of different triplet minima with four different electronic methods.
