# Supplementary material for: Localized and Excimer Triplet Electronic States of Naphthalene Dimers: A Computational Study
Source: Molecules. 2025 Jan 13;30(2):298. doi: 10.3390/molecules30020298 (PMC11767852; doi:10.3390/molecules30020298)
Supplement: Supplementary file 1 [file molecules-30-00298-s001.zip › molecules-3318161-supplementary.pdf]

Localized and excimer triplet electronic states of naphthalene dimers: a computational study.

L. Martinez Fernandez, R. Improta

Supporting Information

Additional computational details

For the calculations performed with ORCA, the RI-J for Coulomb integrals and COSX numerical integration for HF exchange were considered and the Trust Radius Augmented Hessian (TRAH) approach disabled.

Additional Tables and Figures

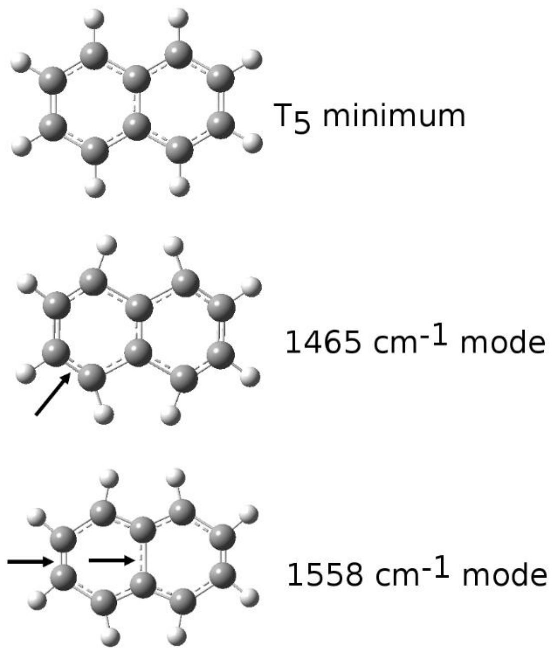

Scheme S1. Schematic depiction of the naphthalene structures distorted according to the motion of the vibrational modes associated to the main vibronic bands of the experimental spectrum. The arrows denote the kind of bonds most involved in the vibrational modes (see text for details).

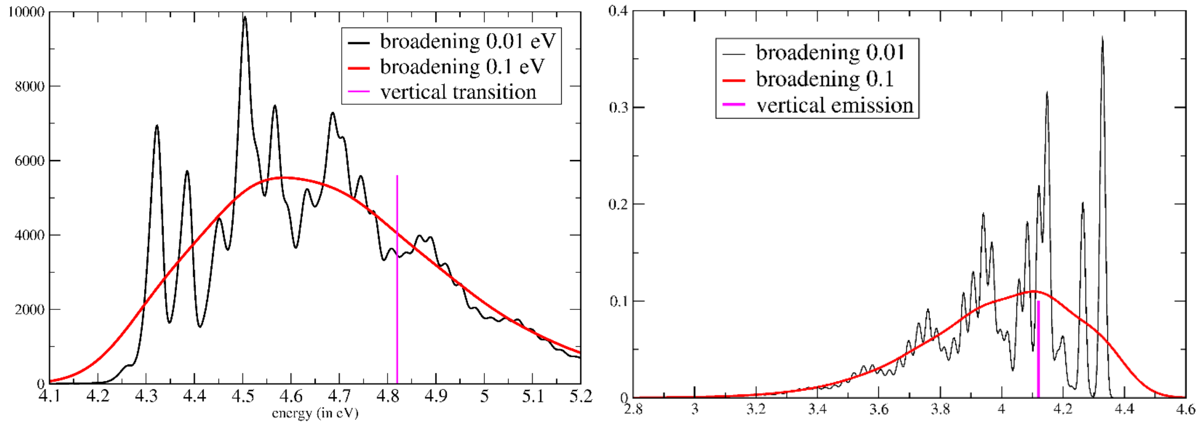

Figure S1. Absorption (left) and emission (right) spectra of the $1B_{2u}$ state computed at the M052-X/6-31+G(d,p) level at 300 K by using two different width for the gaussian used to broad the stick transition.

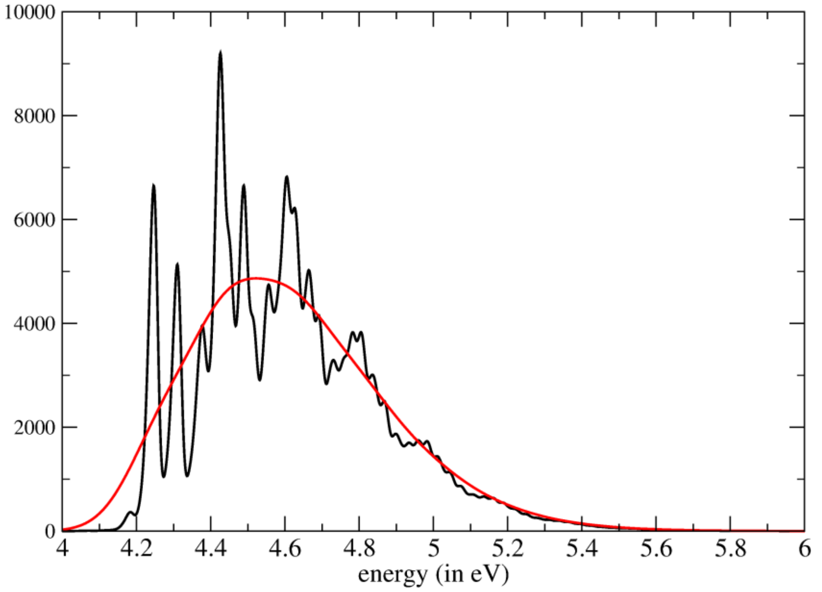

Figure S2. Absorption spectrum of the $1B_{2u}$ state computed at the ωB97X-D /def2tzvp level, at 300 K by using two different width for the gaussian used to broad the stick transition: 0.01 black spectrum, 0.1 red spectrum.

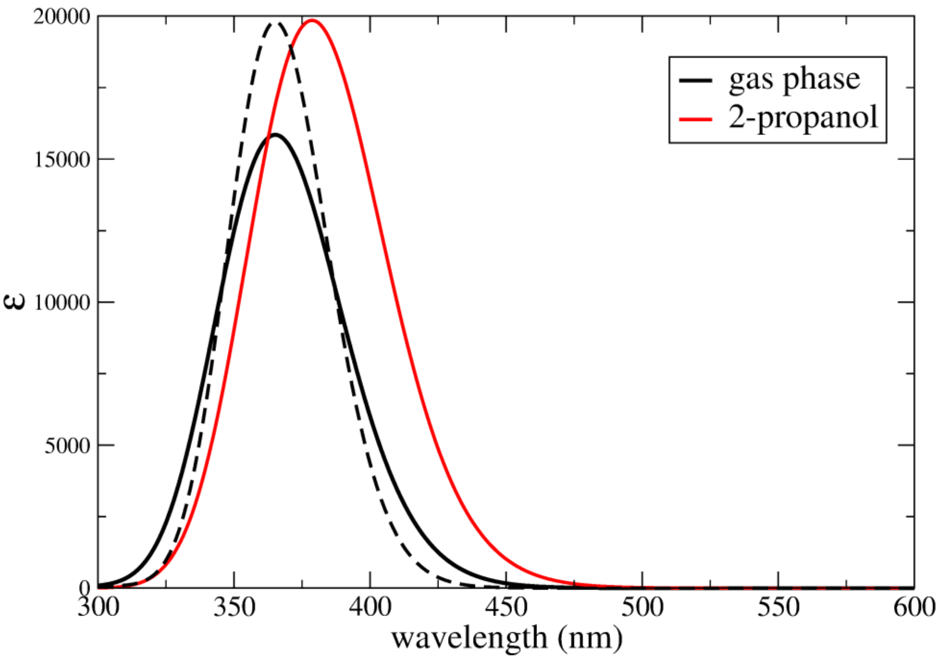

Figure S3. TDA Absorption spectra computed for the T_1 triplet minima of naphthalene ωB97X-D/6-31+G(d,p) level in the gas phase (black) and in 2-propanol (red) by convoluting the electronic stick transition with a gaussian with hwhm=0.25 eV. The gas phase spectrum obtained with a hwhm=0.2 eV is reported as a dashed line.

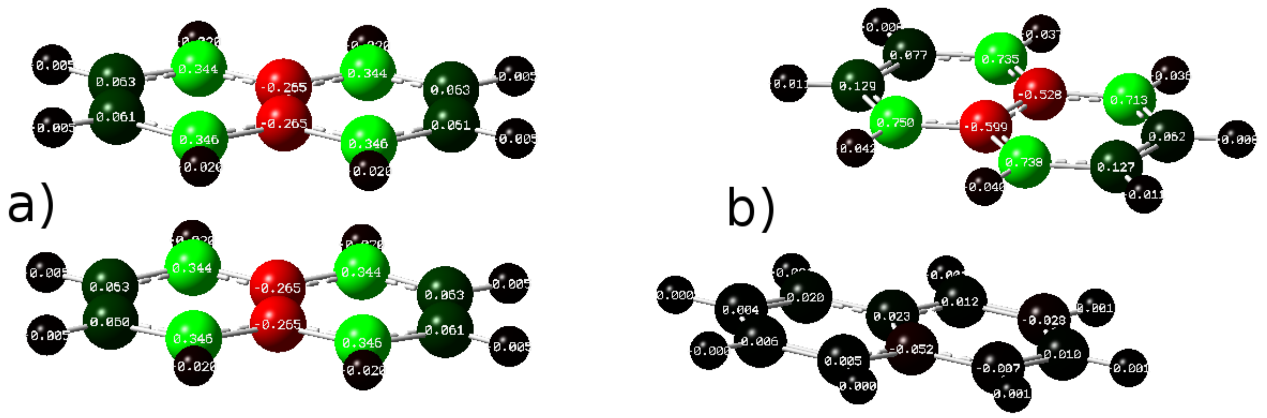

Figure S4. Mulliken spin population computed at the B2LYP-D3/6-31+G(d,p) level for T_1-exc-min (a) and T_1-loc-min (b). Color code: red, negative spin density; green, positive spin density; black, spin density close to zero. The value of the spin density on each atom is reported in white.

Table S1. Main geometrical parameters (in Å) of the lowest energy singlet and triplet minima for naphthalene monomer and dimer, with different coordination geometries (text and Scheme 1). ωB97X-D/6-31+G(d) calculations.

	Monomer		Dimer					
	S_0	T_1	T_1-exc-min		T_1-loc-min		T_1-loc-f2f	
					^{3}N	^{1}N	^{3}N	^{1}N
r_i	1.423	1.452	1.436	1.436	1.451	1.422	1.450	1.423
r_o	1.416	1.358	1.384	1.384	1.358	1.416	1.358	1.413
r_{im}	1.421	1.404	1.411	1.411	1.404	1.420	1.404	1.420
r_{om}	1.372	1.443	1.404	1.404	1.442	1.372	1.440	1.373
d			3.18		3.91		3.48	
η			0.0		-41.3		0.0	

Table S2. Main geometrical parameters (in Å) of the lowest energy singlet and triplet minima for naphthalene monomer and dimer, with different coordination geometries (text and Scheme S1). B2LYP-D3/ 6-31+G(d,p) calculations

	Monomer		Dimer					
	S_0	T_1	T_1-exc-min		T_1-loc-min		T_1-loc-f2f	
					^{3}N	^{1}N	^{3}N	^{1}N
r_i	1.432	1.444	1.438	1.438	1.443	1.432	1.442	1.432
r_o	1.418	1.368	1.390	1.390	1.368	1.417	1.370	1.414
r_{im}	1.422	1.411	1.416	1.416	1.411	1.421	1.411	1.420
r_{om}	1.378	1.439	1.407	1.407	1.438	1.379	1.435	1.380
d			3.17		3.87		3.45	
η			0.0		-43.0		0.0	

Table S3. Relative energy (in eV), with respect to T_1-exc-min, for different T_1-loc stationary points located for the Naphthalene dimer in the gas phase.

	T_1-loc-min		T_1-loc-f2f	T_1-loc-T
	Electronic Energy	Free Energy	Electronic Energy	Electronic Energy
B2LYP-D3/cc-pvtz	+0.09[c]			+0.19
B2PLYP-D3/aug-cc-pvtz	+0.12		+0.08	
M052-X/6-31+G(d,p)	-0.32	-0.54	-0.27	-0.28
M052-X/aug-cc-pvtz	-0.31	-0.60	-0.26	
PBEQIDH/gen	-0.04	-0.19		

Table S4. Vertical absorption energies (VAE in eV) and oscillator strength (f) , computed for different T_1 minima the Naphthalene dimer in the gas phase, according to different methods. TDA calculations.

	B2PLYP-D3/ 6-31+G(d,p)		ωB2PLYP/ 6-31+G(d,p)		ωB97X-D/ 6-31+G(d,p)		ωB97X-D/ def2tzvp	
	VAE	f	VAE	f	VAE	f	VAE	f
T_1-exc-min								
T2	0.69	0.0001	0.45	0.0000	0.532	0.000	0.525	0.000
T3	1.29	0.0000	1.44	0.0000	1.347	0.000	1.347	0.000
T4	1.56	0.0000	1.69	0.0027	1.574	0.000	1.568	0.000
T5	1.63	0.0021	1.75	0.0000	1.671	0.000	1.692	0.000
T6	**2.34**	**0.8615**	**2.11**	**0.7664**	**2.553**	**0.767**	**2.537**	**0.733**
T7	2.43	0.0001	**2.60**	**0.2678**	2.576	0.000	2.578	0.000
T8	**2.50**	**0.2303**	2.72	0.0000	**2.608**	**0.150**	**2.618**	**0.143**
T9	2.62	0.0003	2.93	0.0001	2.791	0.000	2.788	0.000
T10	2.70	0.0001	3.03	0.0001	3.080	0.000	3.128	0.000
T11	2.86	0.0000	3.11	0.0000	3.113	0.000	3.268	0.000
T_1-loc-min								
T2	1.67	0.0003	1.90	0.0001	1.733	0.000	1.761	0.000
T3	1.73	0.0000	2.06	0.0000	1.812	0.000	1.832	0.000
T4	2.02	0.0038	2.66	0.0001	2.196	0.000	2.205	0.000
T5	2.09	0.0021	**3.19**	**0.2591**	2.520	0.000	2.547	0.000
T6	2.13	0.0003	3.22	0.0003	2.635	0.006	2.661	0.005
T7	**2.82**	**0.1317**	3.27	0.0368	3.152	0.027	3.189	0.025
T8	2.89	0.0439	3.33	0.0109	3.190	0.000	3.228	0.000
T9	2.92	0.0175	3.37	0.0250	3.293	0.015	3.319	0.013
T10	3.03	0.0325	3.50	0.0045	**3.377**	**0.139**	**3.437**	**0.152**
T11	3.08	0.0069	3.82	0.0143	3.456	0.003	3.607	0.016
T_1-loc-f2f								
T2	1.62	0.0000	1.90	0.0000	1.732	0.000	1.779	0.000
T3	1.68	0.0000	2.03	0.0000	1.759	0.000	1.822	0.000
T4	1.80	0.0006	2.62	0.0000	2.132	0.000	2.203	0.000
T5	2.00	0.0000	3.24	0.0102	2.355	0.000	2.443	0.003
T6	**2.09**	**0.1406**	**3.25**	**0.3208**	**2.645**	**0.139**	**2.533**	**0.040**
T7	2.86	0.0001	3.25	0.0000	**3.174**	**0.174**	3.219	0.001
T8	**2.86**	**0.1909**	**3.28**	**0.0496**	3.178	0.000	3.274	0.001
T9	2.87	0.0702	**3.43**	**0.0327**	3.293	0.000	3.329	0.024
T10	2.92	0.0000	3.58	0.0038	3.360	0.000	**3.350**	**0.159**
T11	2.99	0.0002	3.79	0.0003	3.426	0.000	3.506	0.011
T1-t	1.70	0.0000	1.90	0.0000	1.769	0.000	1.802	0.000
T2	1.74	0.0000	2.06	0.0000	1.811	0.000	1.826	0.000
T3	2.14	0.0125	2.66	0.0000	2.271	0.000	2.268	0.000
T4	2.25	0.0000	3.24	0.0001	2.567	0.013	2.620	0.013
T5	2.27	0.0001	**3.27**	**0.3306**	3.199	0.000	3.234	0.000
T6	2.83	0.0004	3.43	0.0231	3.353	0.012	3.388	0.006
T7	**3.02**	**0.2251**	3.46	0.0347	**3.362**	**0.196**	**3.402**	**0.216**
T8	3.03	0.0035	3.58	0.0046	3.438	0.004	3.493	0.005
T9	3.04	0.0902	3.63	0.0003	3.487	0.000	3.532	0.000
T10	3.13	0.0000	3.88	0.0000	3.629	0.006	4.161	0.000
T11	3.29	0.0000	3.96	0.0033	3.894	0.000	4.174	0.000
Monomer								
T5	**3.07**	**0.4041**	3.30	0.4478	**3.395**	**0.289**	**3.445**	**0.287**

Cartesian coordinates of T_1-exc-min, optimized in the gas phase at the B2PLYP-D3/6-31+G(d,p) level

C	-1.233785	1.727770	1.295422
C	-2.449541	1.699653	0.588015
C	-2.449534	1.543262	-0.793686
C	-1.233250	1.415488	-1.487556
C	0.007528	1.673395	0.616990
C	0.006744	1.489021	-0.808892
C	1.249353	1.719529	1.295188
C	2.464752	1.683304	0.587493
C	2.463348	1.526502	-0.794176
C	1.246079	1.406990	-1.487813
C	-1.246681	-1.407363	1.487385
C	-2.463666	-1.527159	0.793281
C	-2.464437	-1.683583	-0.588453
C	-1.248794	-1.718945	-1.295731
C	-0.007054	-1.488176	0.808807
C	-0.007231	-1.672587	-0.617072
C	1.232709	-1.415059	1.488001
C	2.449243	-1.543435	0.794672
C	2.449839	-1.700111	-0.587002
C	1.234380	-1.727383	-1.294989
H	1.253370	1.842798	2.373420
H	-3.386225	1.779830	1.127521
H	-1.236750	1.850175	2.373758
H	-1.233414	1.291561	-2.565452
H	-3.385681	1.510397	-1.338836
H	1.245168	1.283178	-2.565720
H	3.399131	1.487430	-1.339557
H	3.402075	1.758057	1.126668
H	1.232442	-1.291373	2.565925
H	-3.399655	-1.489719	1.338422
H	-1.246184	-1.284487	2.565401
H	-1.252373	-1.842197	-2.373968
H	-3.401525	-1.758718	-1.127990
H	1.237758	-1.850724	-2.373218
H	3.386712	-1.781615	-1.125978
H	3.385147	-1.511267	1.340288

Cartesian coordinates of T_1-loc-min, optimized in the gas phase at the B2PLYP-D3/6-31+G(d,p) level

C	-1.249523	0.460394	-0.972013
C	-2.272141	0.792896	-0.008826
C	1.684340	-0.071498	0.933769
C	1.846889	-1.190143	0.055052
C	-1.308897	-0.790093	-1.619996
C	-0.219373	1.392699	-1.220111
C	-3.291372	-0.147071	0.251215
C	-2.215133	2.047097	0.636696
C	1.013668	-2.327220	0.228184
C	2.828754	-1.120731	-0.970499
C	0.676362	-0.122879	1.933110
C	2.524092	1.062596	0.766960

C	-2.357750	-1.731185	-1.334798
C	-0.184391	2.663959	-0.545894
C	-1.156760	2.982747	0.362256
C	-3.325712	-1.417705	-0.418703
C	0.048957	-2.351503	1.212655
C	3.620561	-0.000852	-1.116171
C	3.470338	1.099769	-0.234774
C	-0.126883	-1.235009	2.066504
H	0.567669	1.136118	-1.920260
H	-0.537392	-1.045916	-2.337238
H	-2.984800	2.303796	1.356486
H	-4.061957	0.099292	0.973743
H	2.948810	-1.968204	-1.638003
H	1.140898	-3.175562	-0.436793
H	2.398073	1.906647	1.436676
H	0.537310	0.737169	2.579601
H	4.366233	0.038454	-1.902086
H	4.102859	1.972251	-0.353110
H	-0.903914	-1.255385	2.821738
H	-0.590508	-3.218915	1.325688
H	-4.123479	-2.115941	-0.194420
H	0.620317	3.355481	-0.764384
H	-1.145445	3.937452	0.874903
H	-2.370837	-2.684338	-1.850243

Cartesian coordinates of T_1-f2f, optimized in the gas phase at the B2PLYP-D3/6-31+G(d,p) level

C	-0.002659	2.002955	0.349670
C	-0.001835	1.393515	-0.957042
C	0.002528	-1.993441	-0.349440
C	0.001984	-1.409182	0.958636
C	1.235304	2.290314	0.964269
C	-1.241399	2.287268	0.964147
C	1.235331	1.111725	-1.574512
C	-1.238246	1.108643	-1.574632
C	1.243988	-1.133917	1.589368
C	-1.240533	-1.136781	1.589629
C	1.246636	-2.271050	-0.975489
C	-1.241067	-2.273949	-0.975224
C	2.479747	2.017993	0.302761
C	-2.485100	2.011818	0.302523
C	-2.483148	1.432366	-0.938738
C	2.479361	1.438552	-0.938511
C	2.436874	-1.430848	0.962340
C	-2.432850	-1.436514	0.962852
C	-2.433444	-2.009309	-0.330287
C	2.438531	-2.003606	-0.330812
H	-1.247115	2.744627	1.947893
H	1.239809	2.747615	1.948050
H	-1.241236	0.643010	-2.553579
H	1.239567	0.646109	-2.553461
H	-1.236977	-0.690068	2.578291
H	1.239606	-0.687235	2.578043

H	-1.240079	-2.715416	-1.966966
H	1.246468	-2.712513	-1.967233
H	-3.373958	-1.224019	1.456494
H	-3.375148	-2.236655	-0.817032
H	3.380659	-2.228744	-0.817766
H	3.377584	-1.216167	1.455791
H	3.410195	1.217441	-1.446884
H	-3.416852	2.257645	0.798086
H	-3.413380	1.208930	-1.447195
H	3.410836	2.266126	0.798419

Cartesian coordinates of T_1-T optimized in the gas phase at the B2PLYP-D3/6-31+G(d,p) level

C	-2.504284	0.004611	-0.708229
C	-2.518172	-1.231270	-1.390547
C	-2.494550	-2.480930	-0.677369
C	-2.457818	-2.484526	0.690805
C	-2.442909	-1.238743	1.410951
C	-2.464859	0.000740	0.735566
H	-2.539287	-1.233236	-2.474534
H	-2.503632	-3.409878	-1.234704
H	-2.437012	-3.416408	1.242897
H	-2.405087	-1.246619	2.494430
C	-2.516304	1.244144	-1.383945
C	-2.491460	2.489977	-0.664108
C	-2.453982	2.486224	0.704034
C	-2.439920	1.236582	1.417504
H	-2.538147	1.251922	-2.467894
H	-2.500427	3.421884	-1.216485
H	-2.432495	3.415114	1.261112
H	-2.401759	1.238648	2.500998
C	3.195297	-0.006588	0.000805
C	1.763294	0.001219	-0.028062
C	3.875063	-1.254993	0.016577
C	3.888713	1.234307	0.013011
C	3.198490	2.428301	-0.002712
C	1.781352	2.434308	-0.031670
C	1.079758	1.247325	-0.044174
C	3.171819	-2.441406	0.004504
C	1.754689	-2.431946	-0.023541
C	1.066218	-1.237368	-0.039551
H	1.246826	3.377397	-0.044072
H	3.739833	3.367527	0.006904
H	3.702851	-3.386471	0.016768
H	1.209682	-3.369054	-0.032200
H	4.973734	1.227531	0.034933
H	-0.015622	-1.229863	-0.059106
H	4.960098	-1.260024	0.038324
H	-0.002159	1.251719	-0.066470
